# Improving ductal carcinoma in situ classification by convolutional neural network with exponential linear unit and rank-based weighted pooling

**DOI:** 10.1007/s40747-020-00218-4

**Published:** 2020-11-22

**Authors:** Yu-Dong Zhang, Suresh Chandra Satapathy, Di Wu, David S. Guttery, Juan Manuel Górriz, Shui-Hua Wang

**Affiliations:** 1 School of Informatics, University of Leicester, Informatics Building, University Road, Leicester, LE1 7RH UK; 2 Department of Information Systems, Faculty of Computing and Information Technology, King Abdulaziz University, Jeddah, 21589 Saudi Arabia; 3 School of Computer Engg, KIIT Deemed to University, Bhubaneswar, India; 4 Leicester Cancer Research Center, University of Leicester, Leicester, LE1 7RH UK; 5 University of Melbourne, Melbourne, VIC 3010 Australia; 6 Department of Signal Theory, Networking and Communications, University of Granada, Granada, Spain; 7 School of Architecture Building and Civil Engineering, Loughborough University, Loughborough, LE11 3TU UK

**Keywords:** Ductal carcinoma in situ, Thermal images, Deep learning, Convolutional neural network, Breast thermography, Exponential linear unit, Rank-based weighted pooling, Data augmentation, Color jittering, Visual question answering

## Abstract

Ductal carcinoma in situ (DCIS) is a pre-cancerous lesion in the ducts of the breast, and early diagnosis is crucial for optimal therapeutic intervention. Thermography imaging is a non-invasive imaging tool that can be utilized for detection of DCIS and although it has high accuracy (~ 88%), it is sensitivity can still be improved. Hence, we aimed to develop an automated artificial intelligence-based system for improved detection of DCIS in thermographs. This study proposed a novel artificial intelligence based system based on convolutional neural network (CNN) termed CNN-BDER on a multisource dataset containing 240 DCIS images and 240 healthy breast images. Based on CNN, batch normalization, dropout, exponential linear unit and rank-based weighted pooling were integrated, along with L-way data augmentation. Ten runs of tenfold cross validation were chosen to report the unbiased performances. Our proposed method achieved a sensitivity of 94.08 ± 1.22%, a specificity of 93.58 ± 1.49 and an accuracy of 93.83 ± 0.96. The proposed method gives superior performance than eight state-of-the-art approaches and manual diagnosis. The trained model could serve as a visual question answering system and improve diagnostic accuracy.

## Introduction

Ductal carcinoma in situ (DCIS), also named intra-ductal carcinoma is a pre-cancerous lesion of cells that line the breast milk ducts, but have not spread into the surrounding breast tissue. DCIS is considered the earliest stage of breast cancer (Stage 0) [1], and although cure rates are high the patients still need to be treated, since DCIS can become invasive. Note that there are four other stages: Stage 1 describes invasive breast cancer, the cancer cells of which are invading normal surrounding breast tissues. Stages 2 and 3 describe breast cancers that have invaded regional lymph nodes and Stage 4 represents metastatic cancer which spreads beyond the breast and regional lymph nodes to other distant organs [2]. Upon diagnosis of DCIS, treatment options include breast-conserving surgery (BCS), usually in combination with radiation therapy [3] or mastectomy.

Breast thermography (BT) is an alternative imaging tool to mammography, which is the traditional diagnostic tool for DCIS. Unlike mammography (which uses ionizing radiation to generate an image of the breast), BT utilizes infra-red (IR) images of skin temperature to assist in the diagnosis of numerous medical conditions, and has been suggested to detect breast cancer up to 10 years earlier than mammography [4]. Furthermore, due to its use of ionizing radiation, mammography can increase the risk of breast cancer by 2% with each scan [5].

Automatic interpretation of DCIS [6] by BT images consists of three phases: (1) segmentation of the region of interest, separating the breast from the image; (2) feature extraction, choosing distinguishing features that can help recognize the suspicious lesion; (3) classification, identifying the image as DCIS or healthy.

Previous studies have developed a number of effective artificial intelligence (AI) methods for DCIS detection using BT. Milosevic et al. [7] utilized 50 IR breast images to develop a co-occurrence matrix (COM) and run length matrix (RLM) as IR image descriptors. In the classification stage, a support vector machine (SVM) and naive Bayesian classifier (NBC) were used. Their methods are abbreviated as CRSVM and CRNBC. In addition, Nicandro et al. [8] employed NBC, whereas Chen [9] utilized wavelet energy entropy (WEE) as features to classify breast cancers with promising results. Zadeh et al. [10] combined self-organizing map and multilayer perceptron abbreviated as SMMP and Nguyen [11] introduced Hu moment invariant (HMI) to detect abnormal breasts. Finally, Muhammad [12] combined statistical measure and fractal dimension (SMFD), and Guo [13] proposed a wavelet energy support vector machine (WESVM) to detect breast cancer.

Nevertheless, the above methods require laborious feature engineering (FE), i.e., using domain knowledge to extract features from raw data. To help create an improved, automated AI model quickly and effectively, we proposed to use recent deep learning (DL) technologies, viz, convolutional neural networks (CNNs), which are a broad AI technique combining artificial intelligence and representation learning (RL).

Our contributions lie in four parts: (1) we proposed a novel 5-layer CNN; (2) we introduced exponential linear unit to replace traditional rectified linear unit; (3) we introduced rank-based weighted pooling to replace traditional pooling methods and (4) we used data augmentation to enhance the training set, so as to improve the test performance.

## Background

Table 14 in “Appendix A” gives the abbreviations and their explanations for ease of reading.

### Physical fundamentals

BT is a sub-science field within IR imaging sciences. IR cameras detect radiation in the long IR range (9–14 µm), with the thermal images generated being dubbed thermograms. Physically, Planck’s law stated the spectral of a body for frequency $\omega$ at absolute temperature *T* is given as

1a $$ B\left( {\omega ,T} \right) = \frac{{2o\omega^{3} }}{{l_{s}^{2} }} \times \frac{1}{{\theta \left( {\omega ,T} \right)}} $$

1b $$ \theta \left( {\omega ,T} \right) = \exp \left( {\frac{o\omega }{{k_{{\text{B}}} T}}} \right) - 1, $$

where *B* stands for the spectral radiance, $o$ the Planck constant, *k*_B_ the Boltzmann constant, and $l_{s}$ the light speed,. If replacing frequency $\omega$ by wavelength *λ* using $l_{s} = \lambda \omega$, above equation can be written as:

2a $$ B\left( {\lambda ,T} \right) = \frac{{2ol_{s}^{2} }}{{\lambda^{5} }} \times \frac{1}{{\theta \left( {\lambda ,T} \right)}} $$

2b $$ \theta \left( {\lambda ,T} \right) = \exp \left( {\frac{{ol_{s} }}{{\lambda k_{{\text{B}}} T}}} \right) - 1. $$

Both charge-coupled device (CCD) and complementary metal-oxide-semiconductor (CMOS) sensors in optical cameras detect visible light, and even near-infra-red (NIR) by utilizing parts of the IR spectrum. Basically, they could produce true thermograms with temperatures beyond 280 °C.

In our breast thermogram cases, the thermal imaging cameras have a range of 15–45 °C, and a sensitivity around 0.05 °C. Furthermore, three emitted components (ECs) help generate the following breast thermogram images: (1) EC of the breast, (2) EC of the surrounding medium, and (3) EC in the neighboring tissue.

### Physiological fundamentals

**Fig. 1:**
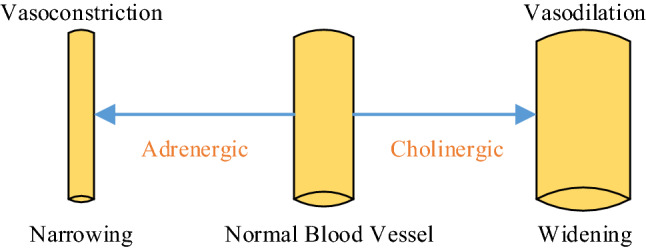
Difference between VC and VD

In healthy tissue, the major regulation and control of dermal circulation is neurovascular, i.e. through the sympathetic nervous system. Its sympathetic response includes both adrenergic and cholinergic. The former causes vasoconstriction (VC, narrowing of blood vessels); conversely, the latter leads to vasodilation (VD, widening of blood vessels). The difference between VC and VD is presented in Fig. 1.

In the early stages of cancer growth, cancer cells produce nitric oxide (NO), resulting in VD. Tumor cells then initiate angiogenesis, which is necessary to sustain breast tumor growth. Both VD and angiogenesis lead to increased blood flow; therefore, the increased heat released as a result of increased blood flow to the tumor results in hotter areas than healthy skin.

The thermogram of a healthy person is symmetrical across the midline. Asymmetry in the thermogram might signify an abnormality, or even a tumor. Therefore, the thermogram illustrates the status of the breast and presence of breast diseases by identifying asymmetric temperature distribution.

Despite this, previous studies [7, 8, 14] have not measured asymmetry directly. As an alternative, those papers employed texture or statistical measures. As a result, this study did not use asymmetry information, and treated each side image (left breast or right breast) as individual images.

### Dataset and preprocessing

240 DCIS breast images and 240 healthy breast (HB) images were obtained from 5 sources: (1) our previous study [12] and further collections after its publication. (2) Ann Arbor thermography [15]; (3) The Breast Thermography Image dataset [16]; (4) The Database for Mastology Research with Infrared Image [17] and (5) online resources using search engines including Google, Yahoo, etc.

Since our dataset is multi-source, we normalized all the collected images using preprocessing techniques. These included: (I) crop: remove background contents and only preserve the breast tissue and (II) resize: all images were re-sampled to the size of $\left[ {128 \times 128 \times 3} \right]$. Suppose original image is $x_{1} \left( t \right),t \in \left[ {1,480} \right]$. After Step I, we have

3 $$ x_{2} \left( t \right) = {\text{Crop}}\left[ {x_{1} \left( t \right),\left( {l_{t} ,r_{t} ,t_{t} ,b_{t} } \right)} \right] $$

where $\left( {l_{t} ,r_{t} ,t_{t} ,b_{t} } \right)$ are four parameters denotes left, right, top, and bottom margins of *t*-th image to be cropped.

Finally, after Step II, we have all the images $x_{3} \left( t \right) \in X_{3}$

4 $$ x_{3} \left( t \right) = {\text{resize}}\left[ {x_{2} \left( t \right),\left( {128,128,3} \right)} \right]. $$

Note some BT images used different pseudo colormaps (PCMs). For example, some used yellow to denote high temperature while some used red; conversely, some used blue to denote low temperature while some used green. We did not apply the same PCM to all BT images within our dataset for four reasons: (1) we expected our AI model would learn to determine a diagnosis based on color difference, not the color itself; (2) humans can make a diagnosis regardless of the PCM configuration, so we believed AI can do the same; (3) we expected our AI model can be universal, i.e., PCM-independent and (4) mixing of PCM color schemes in the training set can help make our AI model more robust when analyzing the test set, i.e., it does not require a particular PCM scheme.

**Fig. 2:**
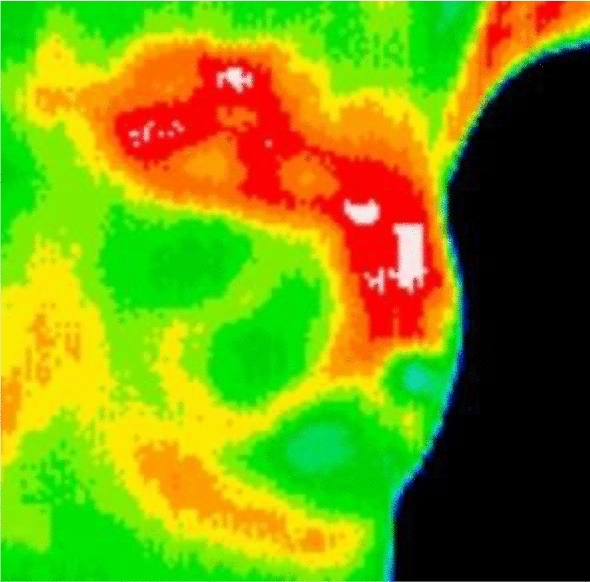
Sample of our dataset

Figure 2 shows a DCIS case, where we can clearly see the temperature difference of the lesion and the surrounding healthy tissues. All the images included in our dataset were checked by agreement of two professional radiologists $\left( {R_{1} ,R_{2} } \right)$ with more than 10 years of experience. If their decisions $\left[ {H\left( {R_{1} } \right),H\left( {R_{2} } \right)} \right]$ agreed, then the images were labelled correspondingly, otherwise, a senior radiologist $\left( {R_{3} } \right)$ was consulted to achieve a consensus:

5 $$ H\left[ {x_{3} \left( t \right)} \right] = \left\{ {\begin{array}{*{20}l}  {H\left[ {x_{3} \left( t \right),R_{1} } \right]} \hfill &\quad {H\left[ {x_{3} \left( t \right),R_{1} } \right] = H\left[ {x_{3} \left( t \right),R_{2} } \right]} \hfill \\  {M\left\{ {H\left[ {x_{3} \left( t \right),\left( {R_{1} ,R_{2} ,R_{3} } \right)} \right]} \right\}} \hfill &\quad {{\text{otherwise}}} \hfill \\  \end{array} } \right.. $$

Here $H$ is the labelling result, $M$ denotes the majority voting, $H\left[ {x_{3} \left( t \right),\left( {R_{1} ,R_{2} ,R_{3} } \right)} \right]$ denotes the labelling results by all three radiologists.

## Methodology

### Improvement 1: exponential linear unit

The activation function mimics the influence of an extra-cellular field on a brain axon/neuron. The real activation function for an axon is quite complicated, and can be written as

6 $$ f_{n} = \frac{1}{c}\left( {\frac{{V_{{n - 1}}^{e} - V_{n}^{e} }}{{\frac{{R_{{n - 1}} }}{2} + \frac{{R_{n} }}{2}}} + \frac{{V_{{n + 1}}^{e} - V_{n}^{e} }}{{\frac{{R_{{n + 1}} }}{2} + \frac{{R_{n} }}{2}}} + \cdots } \right), $$

where *n* means the index of axon’s compartment model, *c* the membrane capacity, $R_{n}$ the axonal resistance of compartment *n*, $V_{n}^{e}$ the extra-cellular voltage outside compartment n relative to the ground [18]. This is difficult to determine in an “artificial neural network”, and thus AI scientists designed some simplistic and ideal activation functions (AFs), which have no direct connection with the axon’s activating function, but those AFs work well for ANNs [19].

An important property of AF is nonlinearity. The reason is stacks of linear function will also be linear, and those kinds of linear AFs can only solve trivial problems and cannot make decisions. Only nonlinear AF can allow neural networks to solve non-trivial problems, such as decision-making. Similar ideas were mentioned as “even our mind is governed by the nonlinear dynamics of complex systems” by Mainzer [20].

Suppose the input is *t*, traditional rectified linear unit (ReLU) [21] $f_{{{\text{ReLU}}}}$ is defined as

7 $$ f_{{{\text{ReLU}}}} \left( t \right) = \max \left( {0,t} \right), $$

with its derivative as

8 $$ f_{{{\text{ReLU}}}}^{\prime } \left( t \right) = \left\{ {\begin{array}{*{20}l}  0 \hfill &\quad {t \le 0} \hfill \\  0 \hfill &\quad {t > 0} \hfill \\  \end{array} } \right.. $$

When $t < 0$, the activation of $f_{{{\text{ReLU}}}}$ values are set to zero, so ReLU cannot train the networks via gradient-based learning. Clevert et al. [22] proposed the exponential linear unit (ELU)

9 $$ f_{{{\text{ELU}}}} \left( {\gamma ,t} \right) = \left\{ {\begin{array}{*{20}l}  {\gamma \left( {e^{t} - 1} \right)} \hfill &\quad {t \le 0} \hfill \\  t \hfill &\quad {t > 0} \hfill \\  \end{array} } \right.. $$

ELU’s derivative is

10 $$ f_{{{\text{ELU}}}}^{\prime } \left( {\gamma ,t} \right) = \left\{ {\begin{array}{*{20}l}  {f_{{{\text{ELU}}}} \left( {\gamma ,t} \right) + \gamma } \hfill &\quad {t \le 0} \hfill \\  t \hfill &\quad {t > 0} \hfill \\  \end{array} } \right. $$

**Fig. 3:**
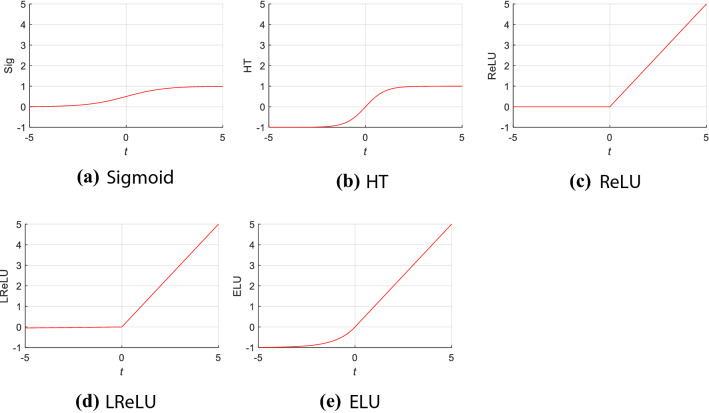
Shape of five different activation functions. *HT* hyperbolic tangent, *ReLU* rectified linear unit, *LReLU* leaky rectified linear unit, *ELU* exponential linear unit)

The default value of $\gamma = 1$. Figure 3 represents the shapes of five different but common AFs. Each subplot has the same range on the *x*-axis and *y*-axis for easy comparison. Information regarding the three AFs (Sigmoid, HT, and LReLU) can be found in “Appendix B”.

### Improvement 2: rank-based weighted pooling

The activation maps (AMs) after conv layer are usually too large, i.e., the size of their width, length, and channels are too large to handle, which will cause (1) overfitting of the training set and (2) large computational costs. Instead pooling layer (PL) is a form of nonlinear downsampling (NLDS) used to solve the above issue. Further, PL can provide invariance-to-translation properties to the AMs.

For a $2 \times 2$ region, suppose the pixels within the region $\Phi = \left\{ {\varphi_{ij} } \right\},\left( {i = 1,2,j = 1,2} \right)$ are

11 $$ \Phi = \left[ {\begin{array}{*{20}l}  {\varphi _{{1,1}} } \hfill &\quad {\varphi _{{1,2}} } \hfill \\  {\varphi _{{2,1}} } \hfill &\quad {\varphi _{{2,2}} } \hfill \\  \end{array} } \right]. $$

Strided convolution (SC) can be regarded as a convolution followed by a special pooling. If the stride is set to 2, the output of SC is:

12 $$ y_{\Phi }^{{{\text{SC}}}} = \varphi_{1,1} . $$

The shortcoming of SC is that it will miss stronger activations if $\varphi_{1,1}$ is not the strongest activation. The advantage of SC is the convolution layer only needs to calculate 1/4 of all outputs in this case, so it can save computation.

L2P calculates the $l_{2}$ norm [23] of a given region $\Phi$. Assume the output value after NLDS is *y*, L2P output $y_{\Phi }^{{{\text{L}}2{\text{P}}}}$ is defined as $y_{\Phi }^{{{\text{L}}2{\text{P}}}} = {\text{sqrt}}\left( {\mathop \sum \nolimits_{i,j = 1}^{2} \phi_{ij}^{2} } \right)$. In this study, we add a constant $1/\left| \Phi \right|$, where $\left| \Phi \right|$ means the number of elements of region $\Phi$. Here $\left| \Phi \right| = 4$ if we use a $2 \times 2{ }$ NLDS pooling. This added new constant 1/4 does not influence training and inference.

13 $$ y_{\Phi }^{{{\text{L}}2{\text{P}}}} = \sqrt {\frac{{\mathop \sum \nolimits_{i,j = 1}^{2} \phi_{ij}^{2} }}{\left| \Phi \right|}} . $$

The average pooling (AP) calculates the mean value in the region $\Phi$ as

14 $$ \begin{aligned}  y_{\Phi }^{{{\text{AP}}}} &= {\text{average}}\left( \Phi \right) \hfill \\  &= \frac{{\varphi _{{1,1}} + \varphi _{{1,2}} + \varphi _{{2,1}} + \varphi _{{2,2}} }}{{\left| \Phi \right|}}. \hfill \\ \end{aligned} $$

The max pooling (MP) operates on the region $\Phi$ and selects the max value. Note that L2P, AP and MP work on every slice separately.

15 $$ \begin{aligned}  y_{\Phi }^{{{\text{MP}}}} &= \max \left( \Phi \right) \hfill \\  &= \max _{{i,j = 1}}^{2} \varphi _{{i,j}} . \hfill \\ \end{aligned} $$

Rank-based weighted pooling (RWP) was introduced to overcome the down-weight (DW), overfitting, and lack of generation (LG) caused by the above pooling methods (L2P, AP, and MP). Instead of computing the $l_{2}$ norm, average, or the max, the output of the RWP $y_{\Phi }^{{{\text{RWP}}}}$ is calculated based on the rank matrix.

First, rank matrix (RM) $R = \left\{ {r_{m} } \right\}$ is calculated based on the values of each element $\varphi_{m} \in \Phi$, usually lower ranks $r \in R$ are assigned to higher values ($\varphi$) as

16 $$ \varphi_{m1} \left\langle {\varphi_{m2} \Rightarrow r_{m1} } \right\rangle r_{m2} . $$

In case of tied values ($\varphi_{m1} = \varphi_{m2}$), a constraint is added as

17 $$ \left( {\varphi_{m1} = \varphi_{m2} } \right) \wedge \left( {m1 > m2} \right) \Rightarrow r_{m1} > r_{m2} . $$

Second, (ER) map $E = \left\{ {e_{m} } \right\}$ is defined as

18 $$ e_{m} = \alpha \times \left( {1 - \alpha } \right)^{{r_{m} - 1}} , $$

where *α* is a hyper-parameter. $\alpha = 0.5$ for all RWP layers, so we do not need to tune $\alpha$ in this study. Equation (18) can be updated as

19 $$ e_{m} = \alpha \times \alpha^{{r_{m} - 1}} = \alpha^{{r_{m} }} . $$

Third, RWP [24] is defined as the summation of $\varphi_{ij}$ and $e_{ij}$ as below

20 $$ y_{\Phi }^{{{\text{RWP}}}} = \mathop \sum \limits_{i,j = 1}^{2} \varphi_{ij} \times e_{ij} . $$

Figure 7 in “Appendix C” gives a schematic comparison of L2P, AP, MP, and RWP.

For better understanding, a pseudocode of RWP is presented in Table 1. We suppose there is an activation map $X_{{{\text{AM}}}}$ with size of $\left[ {R,C} \right]$, where $R$ means the number of rows, and $C$ means the number of columns. Note row index is set to $r$ and column index $c$. The RWP output of $X_{{{\text{AM}}}}$ is symbolized as $y^{{{\text{RWP}}}}$ with size of $\left[ {\frac{R}{2},\frac{C}{2}} \right]$. Table 2 itemizes the equations of every pooling methods.

**Table 1:**
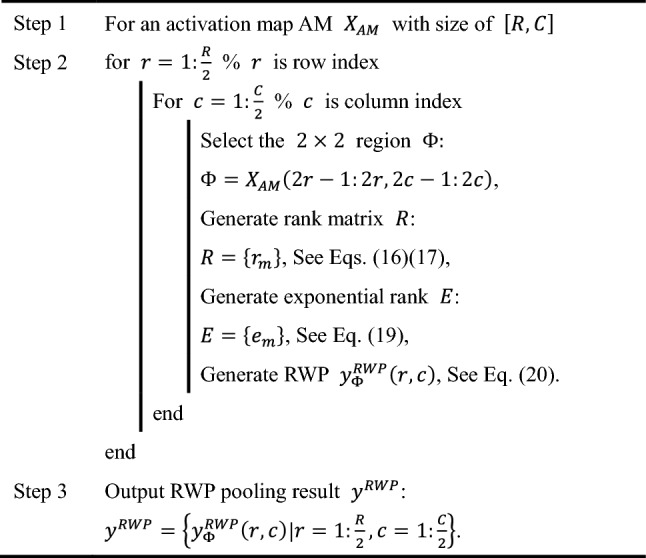
Pseudocode of RWP

**Table 2:** Comparison of different pooling methods

Approach	Output
Raw	$$ \Phi = \left[ {\begin{array}{*{20}l} {\varphi _{{1,1}} } \hfill &\quad {\varphi _{{1,2}} } \hfill \\ {\varphi _{{2,1}} } \hfill &\quad {\varphi _{{2,2}} } \hfill \\ \end{array} } \right] $$
SC	$y_{\Phi }^{{{\text{SC}}}} = \varphi_{1,1}$
L2P	$y_{\Phi }^{{{\text{L}}2{\text{P}}}} = \sqrt {\frac{{\mathop \sum \nolimits_{i,j = 1}^{2} \phi_{ij}^{2} }}{\left| \Phi \right|}}$
AP	$$\begin{array}{*{20}l} {y_{\Phi }^{{{\text{AP}}}} = \frac{{\varphi_{1,1} + \varphi_{1,2} + \varphi_{2,1} + \varphi_{2,2} }}{\left| \Phi \right|}} \\ \end{array}$$
MP	$$\begin{array}{*{20}c} {y_{\Phi }^{{{\text{MP}}}} = \max_{i,j = 1}^{2} \varphi_{i,j} } \\ \end{array}$$
RWP	$y_{\Phi }^{{{\text{RWP}}}} = \mathop \sum \limits_{i,j = 1}^{2} \varphi_{ij} \times e_{ij}$

### Improvement 3: *L*-way data augmentation

Traditional data augmentation is a strategy that enables AI practitioners to radically increase the diversity of training data, without collecting new data actually. In this study, we proposed a *L*-way data augmentation (LDA) technology to further increase the diversity of the training data. The whole preprocessed image set $X_{3}$, from Eq. (4), will separate into $K$ folds:

21 $$ X_{3} \mathop{\longrightarrow}\limits^{{{\text{split}}}}\left\{ {X_{3} \left( {k = 1} \right), \ldots , X_{3} \left( {k = K} \right)} \right\}, $$

where $k$ represents the fold index.

At *k*-th trial, fold *k* will be used as the test set $D_{k}$, and other folds will be used as the training set $C_{k}$:

22a $$ C_{k} = X_{3} - C_{k} $$

22b $$ D_{k} = X_{3} \left( k \right), $$

If we do not consider the index *k*, and just simplify the situations as $X_{3} \to \left\{ {C,D} \right\}$, for each training image $c\left( k \right) \in C,k = 1, \ldots ,\left| C \right|$, we will do the following eight DA techniques. Here we suppose each DA technique will generate $W$ new images.

1. Gamma correction (GC). The equations are defined as:
  23 $$ \begin{aligned}  \overrightarrow {{c^{1} \left( k \right)}} & = {\text{GC}}\left[ {c\left( k \right)} \right] \\   & = \left[ {c_{1}^{{{\text{GC}}}} \left( {k,\eta _{1}^{{{\text{GC}}}} } \right), \ldots c_{W}^{{{\text{GC}}}} \left( {k,\eta _{W}^{{{\text{GC}}}} } \right)} \right], \\ \end{aligned} $$
  where $\eta_{j}^{{{\text{GC}}}} \left( {j = 1, \ldots ,W} \right)$ are GC factors.
2. Rotation. Rotation operation rotates the original image to produce *W* new images [25]:
  24 $$ \begin{aligned} \overrightarrow {{c^{2} \left( k \right)}} &= {\text{RO}}\left[ {c\left( k \right)} \right] \hfill \\ &= \left[ {c_{1}^{{{\text{RO}}}} \left( {k,\eta_{1}^{{{\text{RO}}}} } \right), \ldots c_{W}^{{{\text{RO}}}} \left( {k,\eta_{W}^{{{\text{RO}}}} } \right)} \right] \hfill \\ \end{aligned} $$
  where $\eta_{j}^{{{\text{RO}}}} \left( {j = 1, \ldots ,W} \right)$ are rotation factors.
3. Scaling. All training images $c\left( k \right)$ were scaled [25] as
  25 $$ \begin{aligned} \overrightarrow {{c^{3} \left( k \right)}} &= {\text{SC}}\left[ {c\left( k \right)} \right] \hfill \\ &= \left[ {c_{1}^{{{\text{SC}}}} \left( {k,\eta_{1}^{{{\text{SC}}}} } \right), \ldots c_{W}^{{{\text{SC}}}} \left( {k,\eta_{W}^{{{\text{SC}}}} } \right)} \right], \hfill \\ \end{aligned} $$
  where $\eta_{j}^{{{\text{SC}}}} \left( {j = 1, \ldots ,W} \right)$ are scaling factors.
4. Horizontal shear (HS) transform. $W$ new images were generated by HS transform
  26 $$ \begin{aligned} \overrightarrow {{c^{4} \left( k \right)}} &= {\text{HS}}\left[ {c\left( k \right)} \right] \hfill \\ &= \left[ {c_{1}^{{{\text{HS}}}} \left( {k,\eta_{1}^{{{\text{HS}}}} } \right), \ldots c_{W}^{{{\text{HS}}}} \left( {k,\eta_{W}^{{{\text{HS}}}} } \right)} \right], \hfill \\ \end{aligned} $$
  where $\eta_{j}^{{{\text{HS}}}} \left( {j = 1, \ldots ,W} \right)$ are HS factors.
5. Vertical shear (VS) transform. VS transform was generated similarly to HS transform
  27a $$ \begin{aligned} \overrightarrow {{c^{5} \left( k \right)}} & = {\text{VS}}\left[ {c\left( k \right)} \right] \\ & = \left[ {c_{1}^{{{\text{VS}}}} \left( {k,\eta_{1}^{{{\text{VS}}}} } \right), \ldots c_{W}^{{{\text{VS}}}} \left( {k,\eta_{W}^{{{\text{VS}}}} } \right)} \right], \\ \end{aligned} $$
  27b $$ \eta_{m}^{{{\text{VS}}}} = \eta_{m}^{{{\text{HS}}}} ,\forall m \in 1,2, \ldots ,W. $$
6. Random translation (RT). All training images $c\left( k \right)$ were translated $W$ times with random horizontal shift $\varepsilon^{x}$ and random vertical shift $\varepsilon^{y}$, both values of which are in the range of $\left[ { - \Delta ,\Delta } \right]$, and obey uniform distribution ${\mathcal{U}}$:
  28 $$ \begin{aligned} \overrightarrow {{c^{6} \left( k \right)}}& = {\text{RT}}\left[ {c\left( k \right)} \right] \hfill \\& = \left[ {c_{1}^{{{\text{RT}}}} \left( {k,\varepsilon_{1}^{x} ,\varepsilon_{1}^{y} } \right), \ldots c_{W}^{{{\text{RT}}}} \left( {k,\varepsilon_{W}^{x} ,\varepsilon_{W}^{y} } \right)} \right], \hfill \\ \end{aligned} $$
  where
  29a $$ \varepsilon_{m}^{x} \sim {\mathcal{U}}\left[ { - \Delta ,\Delta } \right],\forall m \in \left[ {1,W} \right] $$
  29b $$ \varepsilon_{m}^{y} \sim {\mathcal{U}}\left[ { - \Delta ,\Delta } \right],\forall m \in \left[ {1,W} \right], $$
  where $\Delta$ is the maximum shift factor.
7. Color jittering (CJ). CJ shifts the color values in original images [26] by adding or subtracting a random value. The advantage of CJ is it can help bring in randomness change to the color channels, so it can aid production of fake color images:
  30 $$ \begin{aligned} \overrightarrow {{c^{7} \left( k \right)}} &= {\text{CJ}}\left[ {c\left( k \right)} \right] \hfill \\ &= \left[ {c_{1}^{{{\text{CJ}}}} \left( {k,\xi_{1}^{r} ,\xi_{1}^{g} ,\xi_{1}^{b} } \right), \ldots c_{W}^{{{\text{CJ}}}} \left( {k,\xi_{W}^{r} ,\xi_{W}^{g} ,\xi_{W}^{b} } \right)} \right]. \hfill \\ \end{aligned} $$
  The shifted color random values are within the range of $\left[ { - \varpi , + \varpi } \right]$, as
  31a $$ \xi_{m}^{{{\text{CC}}}} \sim {\mathcal{U}}\left[ { - \varpi ,\varpi } \right] $$
  31b $$ \forall m \in \left[ {1,W} \right] \wedge \forall {\text{CC}} \in \left\{ {r,g,b} \right\}, $$
  where CC means color channel. $\varpi$ means maximum color shift value.
8. Noise injection. The 0-mean 0.01-variance Gaussian noises [27] were added to all training images to produce $W$ new noised images:
  32 $$ \begin{aligned}  \overrightarrow {{c^{{L/2}} \left( k \right)}} & = {\text{NO}}\left[ {a\left( k \right)} \right] \\   & = \left[ {c_{1}^{{{\text{NO}}}} \left( k \right), \ldots c_{W}^{{{\text{NO}}}} \left( k \right)} \right], \\ \end{aligned} $$
  where NO denotes the noise injection operation.
9. Mirror and concatenation. All the above $L/2$ results are mirrored, we have
  33a $$ \begin{array}{*{20}c} {\overrightarrow {{c^{L/2 + 1} \left( k \right)}} = M\left( {\overrightarrow {{c^{1} \left( k \right)}} } \right)} \\ \end{array} $$
  33b $$ \begin{array}{*{20}c} {\overrightarrow {{c^{L/2 + 2} \left( k \right)}} = M\left( {\overrightarrow {{c^{2} \left( k \right)}} } \right)} \\ \end{array} $$
  $$ \cdots $$
  33c $$ \begin{array}{*{20}c} {\overrightarrow {{c^{L} \left( k \right)}} = M\left( {\overrightarrow {{c^{L/2} \left( k \right)}} } \right)} \\ \end{array} . $$
  where *M* represents the mirror function. All the results are finally concatenated as
  34 $$ \underbrace {{\overrightarrow {{c^{{{\text{LDA}}}} \left( k \right)}} }}_{L \times W + 1} = {\text{concat}}\left\{ {\underbrace {c\left( k \right)}_{1},\underbrace {{\overrightarrow {{c^{1} \left( k \right)}} }}_{W}, \ldots ,\underbrace {{\overrightarrow {{c^{L} \left( k \right)}} }}_{W}} \right\}. $$
  The size of $\overrightarrow {{c^{{{\text{LDA}}}} \left( k \right)}}$ is $L \times W + 1$ images. Thus, the LDA can be regarded as a function $c\left( k \right) \mapsto \overrightarrow {{c^{{{\text{LDA}}}} \left( k \right)}}$.

## Proposed models and algorithm

We proposed five models in total in this study. Table 3 presents their relationships. Model-0 was the base CNN model with $N_{{{\text{CL}}}}$ conv layers and $N_{{{\text{FCL}}}}$ fully connected layers. In Model-0, we used max pooling (MP) and ReLU activation function. Model-1 combined Model-0 with batch normalization (BN) and dropout (DO). Model-2 used ELU to replace ReLU in Model-1, while Model-3 used RWP to replace MP in Model-1. Finally, Model-4 introduced both ELU and RWP to enhance the performance based on Model-1.

**Table 3:** Proposed five models

Index	Inheritance	Name	Description
Model-0	Base CNN model	BCNN	Base model with $N_{{{\text{CL}}}}$ conv layers and $N_{{{\text{FCL}}}}$ fully-connected layers
Model-1	Model-0 + BN + DO	CNN-BD	Add BN and DO to Model-0
Model-2	Model-1 + ELU	CNN-BDE	Use ELU to replace ReLU in Model-1
Model-3	Model-1 + RWP	CNN-BDR	Use RWP to replace MP in Model 1
Model-4	Model-1 + ELU + RWP	CNN-BDER	Use ELU and RWP to replace ReLU and MP in Model-1, respectively

**Fig. 4:**
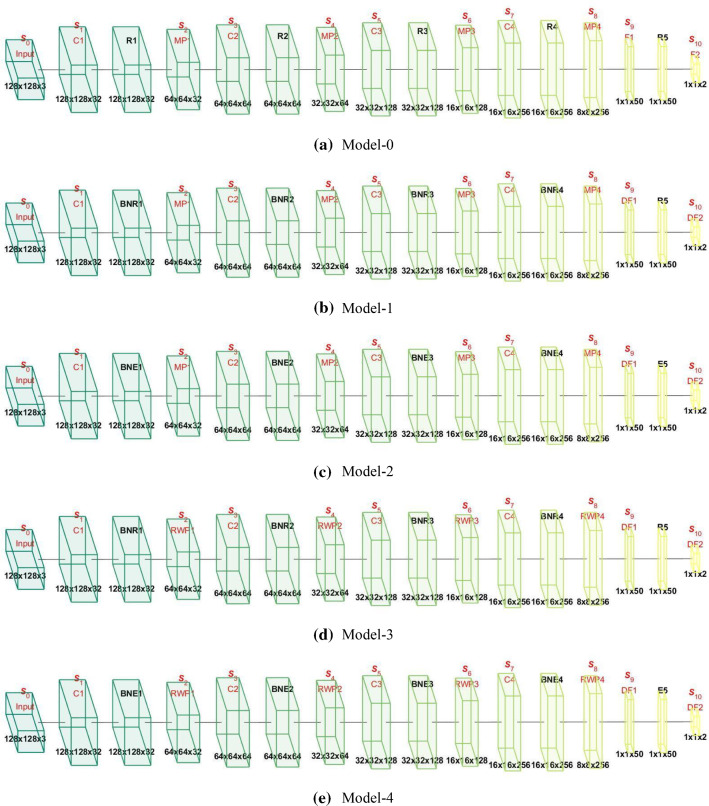
Block chart of five proposed models. *S* size, *C* conv, *BN* batch normalization, *R* *ReLU*, *E* ELU, *D* dropout, *F* fully connected

The top row of Fig. 4a shows the activation maps of the proposed Model-0. Here the size of input was $S_{0} = 128 \times 128 \times 3$, the first conv block is composed of one conv layer, one activation function layer, and one pooling layer. After conv layer, $S_{1} = 128 \times 128 \times 32$. Then after the activation function layer, the output is the same as $S_{1}$. After the pooling layer, the size is $S_{2} = 64 \times 64 \times 32$. The conv block then repeats three times, we have $S_{3} = 64 \times 64 \times 64$ and $S_{4} = 32 \times 32 \times 64$ for the second conv block, $S_{5} = 32 \times 32 \times 128$, and $S_{6} = 16 \times 16 \times 128$ for the third conv block, $S_{7} = 16 \times 16 \times 256$ and $S_{8} = 8 \times 8 \times 256$ for the four conv block. Then $S_{8}$ was flattened and passed through the first fully connected layer with output as $S_{9} = 1 \times 1 \times 50$. The output of the second fully connected layer was $S_{10} = 1 \times 1 \times 2$.

### Measures

The randomness effect of each run reduced performance reliability, so we used $K$-fold cross validation to analyze unbiased performances. The size of each fold is $\left| {X_{3} } \right|/K$. Due to there being two balanced classes (DCIS and HB), each class will have $\left| {X_{3} } \right|/\left( {2 \times K} \right)$ images. The split setting of one trial is shown in Table 6. Within each trial, $\left( {K - 1} \right)$ folds were used as training, and the rest fold were used as test. After combining all $K$ trials, the test image grew to $\left| {X_{3} } \right|$. If above $K$-fold cross validation repeats $Z$ runs, the performance will be reported on $\left| {X_{3} } \right| \times Z$ images.

Suppose the ideal confusion matrix $E^{{{\text{ideal}}}}$ over the test set at *k*-th trial and *z*-th run is

35 $$ E^{{{\text{ideal}}}} \left( {k,z} \right) = \left[ {\begin{array}{*{20}l}  {\frac{{\left| {X_{3} } \right|}}{{2 \times K}}} \hfill &\quad 0 \hfill \\  0 \hfill &\quad {\frac{{\left| {X_{3} } \right|}}{{2 \times K}}} \hfill \\  \end{array} } \right], $$

where the constant 2 is because our dataset is a balanced, i.e., DCIS class has the same size of HB. After combining $K$ trials, the ideal confusion matrix is at $z$-th run is

36 $$ \begin{aligned}  E^{{{\text{ideal}}}} \left( z \right) & = \sum\limits_{{k = 1}}^{K} {E^{{{\text{ideal}}}} } \left( {k,z} \right) \\   & = \left[ {\begin{array}{*{20}l}  {\frac{{\left| {X_{3} } \right|}}{2}} \hfill &\quad 0 \hfill \\  0 \hfill &\quad {\frac{{\left| {X_{3} } \right|}}{2}} \hfill \\  \end{array} } \right] \\ \end{aligned} $$

In realistic inference, we cannot get the perfect diagonal matrix as shown in Eq. (36), suppose the *z*-th run real confusion matrix is

37 $$ \begin{aligned}  E^{{{\text{real}}}} \left( z \right) & = \sum\limits_{{k = 1}}^{K} {E^{{{\text{real}}}} } \left( {k,z} \right) \\   & = \left[ {\begin{array}{*{20}l}  {a\left( z \right)} \hfill &\quad {b\left( z \right)} \hfill \\  {c\left( z \right)} \hfill &\quad {d\left( z \right)} \hfill \\  \end{array} } \right] \\ \end{aligned} $$

where $0 \le a,b,c,d \le \left| {X_{3} } \right|/2$. The four variables $\left( {a,b,c,d} \right)$ represent TP, FN, FP, and TN, respectively. Here *P* means DCIS and *N* means healthy breast (HB).

Four simple measures $\left( {\nu^{1} ,\nu^{2} ,\nu^{3} ,\nu^{4} } \right)$ can be defined as

38a $$ \nu^{1} \left( z \right) = \frac{a\left( z \right)}{{a\left( z \right) + b\left( z \right)}} $$

38b $$ \nu^{2} \left( z \right) = \frac{d\left( z \right)}{{c\left( z \right) + d\left( z \right)}} $$

38c $$ \nu^{3} \left( z \right) = \frac{a\left( z \right)}{{a\left( z \right) + c\left( z \right)}} $$

38d $$ \nu^{4} \left( z \right) = \frac{a\left( z \right) + d\left( z \right)}{{a\left( z \right) + b\left( z \right) + c\left( z \right) + d\left( z \right)}}. $$

where $\left[ {\nu^{1} \left( z \right),\nu^{2} \left( z \right),\nu^{3} \left( z \right),\nu^{4} \left( z \right)} \right]$ means sensitivity, specificity, precision, and accuracy at *z*-th run, respectively. Besides, *F*1 score $\nu^{5} \left( z \right)$, Matthews correlation coefficient (MCC) $\nu^{6} \left( z \right)$, and Fowlkes–Mallows index (FMI) $\nu^{7} \left( z \right)$ can be defined as:

39a $$ \begin{aligned}  \nu ^{5} \left( z \right) & = 2 \times \frac{{\nu ^{3} \left( z \right) \times \nu ^{1} \left( z \right)}}{{\nu ^{3} \left( z \right) + \nu ^{1} \left( z \right)}} \\   & = \frac{{2 \times a\left( z \right)}}{{2 \times a\left( z \right) + b\left( z \right) + c\left( z \right)}} \\ \end{aligned} $$

39b $$ \nu^{6} \left( z \right) = \frac{d\left( z \right) \times a\left( z \right) - c\left( z \right) \times b\left( z \right)}{{\sqrt {\gamma \left( z \right)} }} $$

39c $$ \begin{aligned}  \gamma \left( z \right) & = \left[ {c\left( z \right) + a\left( z \right)} \right] \times \left[ {a\left( z \right) + b\left( z \right)} \right] \\   & \quad \times \left[ {d\left( z \right) + c\left( z \right)} \right] \times \left[ {d\left( z \right) + b\left( z \right)} \right] \\ \end{aligned} $$

39d $$ \nu^{7} \left( z \right) = \sqrt {\frac{a\left( z \right)}{{a\left( z \right) + c\left( z \right)}} \times \frac{a\left( z \right)}{{a\left( z \right) + b\left( z \right)}}} . $$

After averaging $Z$ runs, we can calculate the mean $\left( M \right)$ and standard deviation (SD) of all *k*-th $\left( {\forall k \in \left[ {1,7} \right]} \right)$ measures as

40a $$ M\left( {\nu^{k} } \right) = \frac{1}{Z} \times \mathop \sum \limits_{z = 1}^{Z} \nu^{k} \left( z \right) $$

40b $$ {\text{SD}}\left( {\nu^{k} } \right) = \sqrt {\frac{1}{Z - 1} \times \mathop \sum \limits_{z = 1}^{Z} \left[ {\nu^{k} \left( z \right) - M\left( {\nu^{k} } \right)} \right]^{2} } . $$

The result is reported in the format of $M \pm {\text{SD}}$. For ease of typing, we write it in short as MSD.

## Experiments and results

### Parameter setting

Table 4 shows the parameter setting of variables in this study. The values were obtained using trial-and-error. The total size of our dataset was 480, and thus the size of the preprocessed image set is $\left| {X_{3} } \right| = 480$. The number of folds and runs were all set to 10, i.e., $K = 10, Z = 10$. Then, each fold contained 48 images, that is 24 DCIS and 24 HB images. The training set contained $\left| C \right| = 432$ images, and the test set contained $\left| D \right| = 48$ images. The number of DA ways was $L = 16$, the number of new images for each DA technique was $W = 30$. Thus, we created $L \times W = 480$ new images for every training image. The number of conv layers/blocks was $N_{{{\text{CL}}}} = 4$, and the number of fully connected layers/blocks was $N_{{{\text{FCL}}}} = 2$.

**Table 4:** Parameter setting of variables

Parameter	Meaning	Value
$\left| {X_{3} } \right|$	Size of preprocessed image set	480
$\left| {C_{k} } \right|$	Size of training set at *k*-th trial	432
$\left| {D_{k} } \right|$	Size of test set at *k*-th trial	48
$K$	Total number of *k*-folds	10
$W$	Number of new images for each DA	30
$L$	Number of DA techniques	16
$\varpi$	Maximum color shift value	50
$Z$	Total number of runs of *K*-fold cross validation	10
$N_{{{\text{CL}}}}$	Number of conv layers/blocks	4
$N_{{{\text{FCL}}}}$	Number of fully connected layers/blocks	2

Table 5 itemizes the LDA parameter settings. The GC factors $\eta^{{{\text{GC}}}}$ varied from 0.4 to 1.6 with an increase of 0.04, skipping the value of 1. The rotation vector $\eta^{{{\text{RO}}}}$ was in the value from $- W$ to $W$ an increase of 2°, skipping $\eta^{{{\text{RO}}}} = 0$. Scaling factor $\eta^{{{\text{SC}}}}$ varied from 0.7 to 1.3 with an increase of 0.02, skipping $\eta^{{{\text{SC}}}} = 1$. HS factors $\eta^{{{\text{HS}}}}$ varied from − 0.15 to 0.15 with an increase of 0.01, skipping the value of $\eta^{{{\text{HS}}}} = 0$. The maximum shift factor $\Delta = 15$. The maximum color shift value was $\varpi = 50$.

**Table 5:** LDA parameter setting

LDA parameter	Values
GC factors	$\eta_{1}^{{{\text{GC}}}} = 0.4,\eta_{2}^{{{\text{GC}}}} = 0.44, \ldots ,\eta_{15}^{{{\text{GC}}}} = 0.96$, $\eta_{16}^{{{\text{GC}}}} = 1.04,\eta_{17}^{{{\text{GC}}}} = 1.08, \ldots \eta_{W}^{{{\text{GC}}}} = 1.6$
Rotation factors	$\eta_{1}^{{{\text{RO}}}} = - W^\circ ,\eta_{2}^{{{\text{RO}}}} = - W + 2^\circ , \ldots ,\eta_{15}^{{{\text{RO}}}} = - 2^\circ$, $\eta_{16}^{{{\text{RO}}}} = + 2^\circ ,\eta_{17}^{{{\text{RO}}}} = + 4^\circ , \ldots ,\eta_{W}^{{{\text{RO}}}} = + W^\circ$
Scaling factors	$\eta_{1}^{{{\text{SC}}}} = 0.7,\eta_{2}^{{{\text{SC}}}} = 0.72, \ldots ,\eta_{15}^{{{\text{SC}}}} = 0.98$, $\eta_{16}^{{{\text{SC}}}} = 1.02,\eta_{17}^{{{\text{SC}}}} = 1.04, \ldots ,\eta_{W}^{{{\text{SC}}}} = 1.3$
HS factors	$\eta_{1}^{{{\text{HS}}}} = - 0.15,\eta_{2}^{{{\text{HS}}}} = - 0.14, \ldots ,\eta_{15}^{{{\text{HS}}}} = - 0.01$, $\eta_{16}^{{{\text{HS}}}} = + 0.01,\eta_{17}^{{{\text{HS}}}} = + 0.02, \ldots ,\eta_{W}^{{{\text{HS}}}} = + 0.15$
Maximum shift factor	$\Delta = 15$
Maximum color shift value	$\varpi = 50$

Table 6 shows the *K*-fold cross validation setting, which was used in the experiment to report unbiased performances [28]. For each trial, the training image set contained 216 DCIS and 216 HB images. Then after *L*-way data augmentation, the LDA training set contained 103,896 images for each class, and thus together $\left| {{\text{DA}}\left( C \right)} \right| = 207,792$ images. The size of the test set during each trial was only 48 images. Combining 10 trials, the final combined test set is the same as the original dataset of 480 images.

**Table 6:** *K*-fold cross validation setting

Set	DCIS	HB	Total
Training (ninefolds)	216	216	$\left| C \right| = 432$
LDA training	103,896	103,896	$\left| {{\text{DA}}\left( C \right)} \right| =$ 207,792
Test (onefold)	24	24	$\left| D \right| = 48$
Total	240	240	$\left| {X_{3} } \right| = 480$

### Statistical result of proposed model-4

The ten runs of our Model-4 results are shown in Table 7. Here it shows using our Model-4 CNN-BDER yielded $\nu^{1} = 94.08 \pm { }1.22$, $\nu^{2} = 93.58 \pm { }1.49$, $\nu^{3} = 93.63 \pm { }1.37$, $\nu^{4} = 93.83 \pm { }0.96$, $\nu^{5} = 93.85 \pm { }0.94$, $\nu^{6} = 87.68 \pm { }1.91$, $\nu^{7} = 93.85 \pm { }0.94$. In summary, our model-4 showed high accuracy, potentially aiding radiologists to make fast and accurate decisions.

**Table 7:** 10 runs of the proposed model-4

Run	Sen $\nu^{1}$	Spc $\nu^{2}$	Prc $\nu^{3}$	Acc $\nu^{4}$	F1 $\nu^{5}$	MCC $\nu^{6}$	FMI $\nu^{7}$
1	92.50	94.58	94.47	93.54	93.47	87.10	93.48
2	92.92	92.50	92.53	92.71	92.72	85.42	92.72
3	94.58	93.33	93.42	93.96	94.00	87.92	94.00
4	94.17	95.42	95.36	94.79	94.76	89.59	94.76
5	94.58	93.33	93.42	93.96	94.00	87.92	94.00
6	92.50	93.75	93.67	93.13	93.08	86.26	93.08
7	94.17	90.42	90.76	92.29	92.43	84.64	92.45
8	95.42	95.42	95.42	95.42	95.42	90.83	95.42
9	93.75	94.17	94.14	93.96	93.95	87.92	93.95
10	96.25	92.92	93.15	94.58	94.67	89.22	94.68
MSD	94.08 ± 1.22	93.58 ± 1.49	93.63 ± 1.37	93.83 ± 0.96	93.85 ± 0.94	87.68 ± 1.91	93.85 ± 0.94

### Model comparison

We next compared the Model-4 CNN-BDER result with other four models (Model-0 BCNN, Model-1 CNN-BD, Model-2 CNN-BDE, and Model-3 CNN-BDR). The comparison results are shown in Table 8. Here, Model-4 CNN-BDER yielded the best results among all five models. Note that $\nu^{2}$ and $\nu^{3}$ of Model-3 CNN-BDR are quite close to those of Model-4 CNN-BDER, but considering the results were obtained using an average of ten runs, we can still conclude that Model-4 CNN-BDER has higher accuracy than Model-3 CNN-BDR in terms of all seven indicators.

**Table 8:** Model comparison (with LDA)

Approach	Sen $\nu^{1}$	Spc $\nu^{2}$	Prc $\nu^{3}$	Acc $\nu^{4}$	F1 $\nu^{5}$	MCC $\nu^{6}$	FMI $\nu^{7}$
Model-0	90.54 ± 0.90	91.58 ± 1.65	91.51 ± 1.55	91.06 ± 1.05	91.02 ± 1.01	82.14 ± 2.10	91.02 ± 1.01
Model-1	91.71 ± 2.06	91.96 ± 0.94	91.94 ± 0.95	91.83 ± 1.28	91.81 ± 1.35	83.68 ± 2.55	91.82 ± 1.35
Model-2	93.58 ± 1.66	92.54 ± 1.34	92.63 ± 1.22	93.06 ± 1.09	93.10 ± 1.10	86.15 ± 2.17	93.10 ± 1.10
Model-3	92.83 ± 1.53	93.54 ± 1.39	93.50 ± 1.37	93.19 ± 1.29	93.16 ± 1.31	86.38 ± 2.57	93.16 ± 1.31
Model-4	**94.08 ± 1.22**	**93.58 ± 1.49**	**93.63 ± 1.37**	**93.83 ± 0.96**	**93.85 ± 0.94**	**87.68 ± 1.91**	**93.85 ± 0.94**

Bold means the best

Kruskal–Wallis test was preformed based on Model-4 against Model-(*m*), where $m = 0,1,2,3$. The *p* value result matrix $P$ is listed in Table 9. The null hypothesis is the indicator vector $\nu^{n} \left( {n = 1, \ldots ,7} \right)$ of $Z$ runs of Model-(*m*) and that of Model-4 come from the same distribution, and the alternative hypothesis that not all samples are obtained from the same distribution. Then we recorded the corresponding *p* value as $p\left( {m,n} \right)$. The final matrix $P = \left[ {p\left( {m,n} \right)} \right],m = 0, \ldots ,3,n = 1, \ldots ,7$. Note here we chose $Z = 30$. The reason is our data are not normally distributed (see Table 7), so it is important to obtain a larger sample set.

**Table 9:** *p* value of hypothesis test (*Z* = 30)

*m*	Sen $\nu^{1}$	Spc $\nu^{2}$	Prc $\nu^{3}$	Acc $\nu^{4}$	F1 $\nu^{5}$	MCC $\nu^{6}$	FMI $\nu^{7}$
0	**2.61e−11**	**1.96e−5**	**9.76e−7**	**1.04e−10**	**3.42e−11**	**8.23e−11**	**3.42e−11**
1	**3.03e−6**	**1.46e−5**	**5.21e−6**	**3.19e−8**	**2.91e−8**	**1.72e−8**	**2.45e−8**
2	0.3169	**0.0027**	**0.0017**	**0.0076**	**0.0102**	**0.0069**	**0.0098**
3	**0.0021**	0.7388	0.5740	**0.0388**	**0.0397**	**0.0325**	**0.0397**

Bold means *p* < 0.05

The first row and second row of Table 9 show that all *p* values are < 0.05. So, the test rejects the null hypothesis at the 5% significance level, indicating that Model-4 is significantly better than Model-0 and Model-1 for all seven indicators. For the third row, the *p* values show that Model-4 is significantly better than Model-2 for all indicators other than sensitivity $\nu^{1}$. For the last row, the *p* values show that Model-4 is significantly better than Model-3 for all indicators other than specificity $\nu^{2}$ and precision $\nu^{3}$.

### Effect of LDA

Table 10 presents the results of not using LDA, showing decreased accuracy compared to those using LDA and highlights the effectiveness of our proposed LDA. The future research direction is to explore more types of DA techniques and increase the diversity of LDA, hence, improving the generalization ability of our AI models. Note that Model-0 BCNN and Model-1 CNN-BD without LDA obtain performances lower than 90%, which are worse than traditional AI methods that do not utilize deep learning. This means deep learning with big data can improve performance, if we do not have big data (not using data augmentation means our training set is only 432 images as shown in Table 6), then deep models may not compete with traditional shallow models.

**Table 10:** Results of not using LDA

Approach	Sen $\nu^{1}$	Spc $\nu^{2}$	Prc $\nu^{3}$	Acc $\nu^{4}$	F1 $\nu^{5}$	MCC $\nu^{6}$	FMI $\nu^{7}$
M0-NLDA	89.46 ± 1.16	87.67 ± 1.02	87.89 ± 0.89	88.56 ± 0.73	88.66 ± 0.74	77.15 ± 1.47	88.67 ± 0.74
M1-NLDA	89.75 ± 1.81	89.46 ± 1.42	89.50 ± 1.26	89.60 ± 1.10	89.62 ± 1.14	79.23 ± 2.19	89.62 ± 1.14
M2-NLDA	91.54 ± 1.47	92.04 ± 1.74	92.02 ± 1.63	91.79 ± 1.26	91.77 ± 1.25	83.60 ± 2.52	91.78 ± 1.25
M3-NLDA	91.21 ± 0.75	91.50 ± 1.23	91.49 ± 1.12	91.35 ± 0.74	91.34 ± 0.71	82.72 ± 1.47	91.35 ± 0.71
M4-NLDA	92.17 ± 1.36	91.46 ± 1.41	91.53 ± 1.32	91.81 ± 1.11	91.84 ± 1.10	83.64 ± 2.22	91.84 ± 1.10
M4-LDA	**94.08 ± 1.22**	**93.58 ± 1.49**	**93.63 ± 1.37**	**93.83 ± 0.96**	**93.85 ± 0.94**	**87.68 ± 1.91**	**93.85 ± 0.94**

Bold means the best

*M* model, *NLDA* not using LDA

**Fig. 5:**
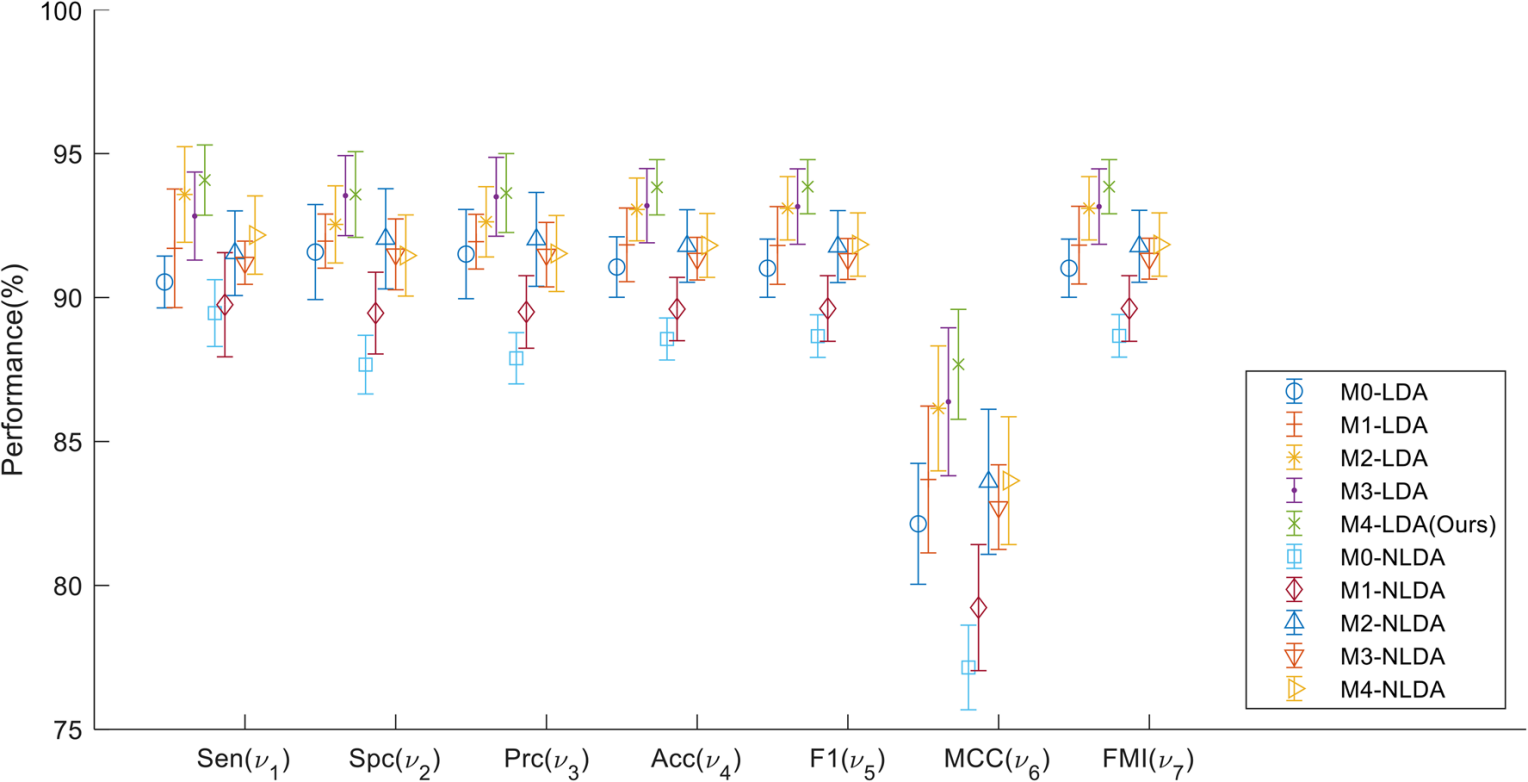
Using LDA versus not using LDA (*M* model, *LDA* using proposed LDA, *NLDA* not using LDA)

Figure 5 summarizes and compares all ten models, where LDA and NLDA represent use and non-use of LDA, respectively. From Fig. 5 we can clearly observe that our Model-4 CNN-BDER using LDA can obtain the best performance among all six models.

Here we do not run hypothesis test, since all the models without LDA show reduced performance than the models with LDA. We have already proven that the statement “Model-4 is better than Models-(0–3)” is statistically significant, so we can conclude that Model-4 is better than Models without LDA.

### Comparison to state-of-the-art approaches

Our proposed Model-4 CNN-BDER was compared with state-of-the-art approaches. First, we used the 40-image dataset in Ref. [12]. The comparison results are presented in Table 11. Note here the performance of our Model-4 differs from previous experiments, because we analyzed a smaller dataset (40-images). The reason why our method is better than SMFD [12] is because SMFD, i.e., statistical measure and fractal dimension, can help extract statistical and global texture information, but it is inefficient in extracting local information.

**Table 11:** Comparison with Ref [12] on 40-image dataset

Method	Sen $\nu^{1}$	Spc $\nu^{2}$	Prc $\nu^{3}$	Acc $\nu^{4}$	F1 $\nu^{5}$	MCC $\nu^{6}$	FMI $\nu^{7}$
SMFD [12]	93.0	92.5	92.54	92.8	92.77	85.50	92.77
Model-4 (ours)	94.50 ± 1.58	94.00 ± 2.11	94.07 ± 1.90	94.25 ± 1.21	94.27 ± 1.18	88.53 ± 2.36	94.28 ± 1.17

Next, we compared our Model-4 with recent state-of-the-art algorithms on the entire 480-image dataset using 10 runs of tenfold cross validation. The comparison algorithms include NBC [8], CRNBC [7], CRSVM [7], WEE [9], SMMP [10], HMI [11], SMFD [12], WESVM [13]. The comparative results are shown in Table 12. Here Ref. [7] provided two methods, one using naive Bayesian classifier, and the other using support vector machine.

**Table 12:** Comparison results on 480-image dataset

Method	Sen $\nu^{1}$	Spc $\nu^{2}$	Prc $\nu^{3}$	Acc $\nu^{4}$	F1 $\nu^{5}$	MCC $\nu^{6}$	FMI $\nu^{7}$
NBC [8]	69.04 ± 1.80	70.33 ± 2.22	69.98 ± 1.25	69.69 ± 0.90	69.49 ± 0.93	39.40 ± 1.78	69.50 ± 0.92
CRNBC [7]	81.33 ± 2.11	84.54 ± 1.77	84.07 ± 1.30	82.94 ± 0.74	82.65 ± 0.90	65.95 ± 1.46	82.68 ± 0.88
CRSVM [7]	81.46 ± 2.12	88.71 ± 1.14	87.85 ± 0.92	85.08 ± 0.78	84.51 ± 0.98	70.38 ± 1.48	84.58 ± 0.95
WEE [9]	90.17 ± 1.47	88.17 ± 1.69	88.43 ± 1.35	89.17 ± 0.51	89.27 ± 0.50	78.38 ± 0.99	89.29 ± 0.49
SMMP [10]	88.17 ± 2.12	89.54 ± 1.69	89.42 ± 1.49	88.85 ± 1.21	88.77 ± 1.27	77.75 ± 2.40	88.78 ± 1.27
HMI [11]	66.46 ± 2.09	76.50 ± 1.55	73.89 ± 0.99	71.48 ± 0.80	69.96 ± 1.15	43.20 ± 1.56	70.07 ± 1.10
SMFD [12]	90.96 ± 0.86	90.63 ± 1.21	90.67 ± 1.10	90.79 ± 0.78	90.81 ± 0.76	81.59 ± 1.57	90.81 ± 0.76
WESVM [13]	75.29 ± 1.86	78.04 ± 1.15	77.43 ± 0.90	76.67 ± 0.95	76.33 ± 1.12	53.37 ± 1.88	76.35 ± 1.12
Model-4 (ours)	94.08 ± 1.22	93.58 ± 1.49	93.63 ± 1.37	93.83 ± 0.96	93.85 ± 0.94	87.68 ± 1.91	93.85 ± 0.94

The results in Table 12 showed that our Model-4 CNN-BDER method performed better than eight state-of-the-art approaches. Except MCC $\nu^{6}$, the other six indicators of our method are greater than 93%. While, the second best method is SMFD [12], whose seven indicator values are all less than 91%. SMFD [12] can help extract statistical and global texture information, but it is inefficient when extracting local information. WEE [9] has a similar problem, since wavelet energy entropy uses wavelet to extract multi-resolution information, and a higher decomposition level of wavelet can extract finer-resolution. But it is difficult to run high-level decomposition in practice. Hence, the information from WEE [9] is mostly at a coarse level. CRNBC [7] and CRSVM [7] used co-occurrence matrix (COM) and run length matrix (RLM) as the feature extraction method, and employed naive Bayesian classifier (NBC) and support vector machine (SVM) as classifiers. COM computes the distribution of co-occurring pixel values at given offsets, while RLM computes the size of homogeneous runs for each grey level. Both features are easy to implement for computer scientists, but their capability of distinguishing tumors from surrounding healthy issues needs to be verified. Also, NBC and SVM are traditional classifiers, whose performances are not as high compared to recent deep learning approaches. SMMP [10] combined self-organizing map (SOM) and multilayer perceptron (MLP) methods. SOM used unsupervised learning to generate a low-dimensional discretized representation of the input space from the training image samples, while MLP has only one hidden layer that may limit its expressivity power. WESVM [13] used wavelet energy support vector machine as the classifier. However, wavelet energy is not a popular feature descriptor, whose improvements and modifications on wavelet energy are still in progress. The two worst methods are NBC [8] and HMI [11]. The former assumes the presence/absence of a feature of a class is unrelated to the presence/absence of any other features; however, this assumption is difficult to fulfil in practice. The latter employed seven Hu moment invariants as feature descriptors, which may be insufficient to capture information regarding breast cancer masses. The performance can be improved by combining with other feature descriptors. In all, Table 12 shows the improved performance of our Model-4 CNN-BDER method.

**Table 13:** Manual diagnosis by three experienced radiologists

Observer	Sen $\nu^{1}$	Spc $\nu^{2}$	Prc $\nu^{3}$	Acc $\nu^{4}$
$P_{1}$	71.67	74.17	73.50	72.92
$P_{2}$	81.25	73.75	75.58	77.50
$P_{3}$	75.42	82.50	81.17	78.96

### Comparison to manual diagnosis

Three experienced radiologists $\left( {P_{1} ,P_{2} ,P_{3} } \right)$ were invited to independently inspect our dataset of 480 thermogram images. None of the radiologists had observed any of the images in advance.

**Fig. 6:**
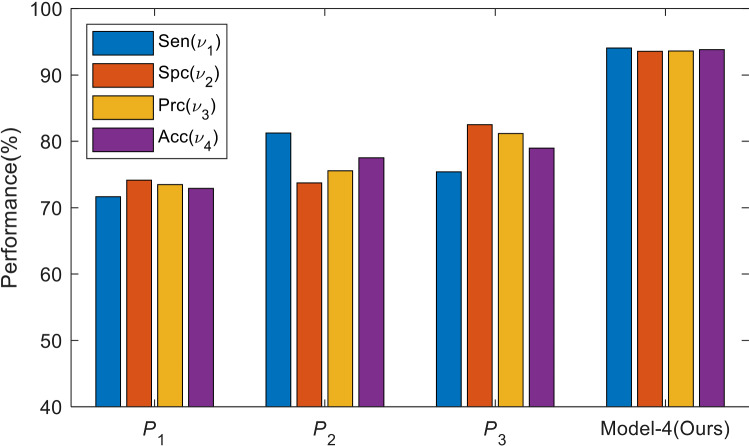
Comparison of proposed model against three radiologists

**Fig. 7:**
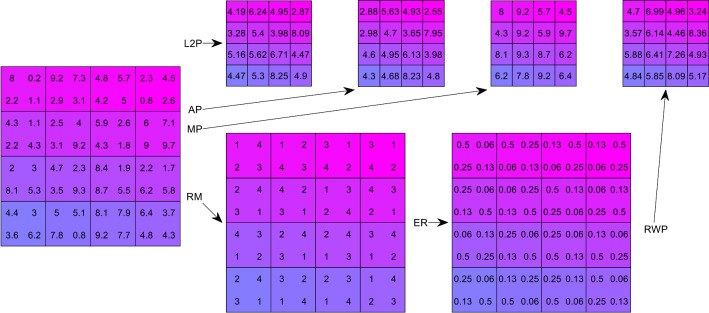
A schematic of L2P, AP, MP, and RWP

The results of three radiologists are itemized in Table 13. The first radiologist $\left( {P_{1} } \right)$ obtained a sensitivity of 71.67%, a specificity of 74.17%, a precision of 73.50%, and an accuracy of 72.92%. The second radiologist $\left( {P_{2} } \right)$ obtained the four indicators as 81.25%, 73.75%, 75.58%, and 77.50%, respectively. The third radiologist $P_{3}$ obtained the four measures as 75.42%, 82.50%, 81.17%, and 78.96%, respectively. Comparing Table 13 with our method Model-4, which is also illustrated in Fig. 6, from which we can see that our proposed CNN-BDER method can give higher performance than manual diagnosis. The reason may be DCIS is Stage 0 of breast cancer, so some lesions are difficult to discern by radiologists while AI can potentially capture those slight and minor lesions.

## Conclusions

We built a new DCIS detection system based on breast thermal images. The method CNN-BDER is based on convolutional neural network, and CNN-BDER has three contributions: (1) use of exponential linear unit to replace traditional ReLU function; (2) use of rank-based weighted pooling to replace traditional max pooling and (3) A *L*-way data augmentation was proposed.

The results show that our Model-4 CNN-BDER method can achieve $\nu^{1} = 94.08 \pm { }1.22$, $\nu^{2} = 93.58 \pm { }1.49$, $\nu^{3} = 93.63 \pm { }1.37$, $\nu^{4} = 93.83 \pm { }0.96$, $\nu^{5} = 93.85 \pm { }0.94$, $\nu^{6} = 87.68 \pm { }1.91$, $\nu^{7} = 93.85 \pm { }0.94$. Our Model-4 offers improved performance over not only the other four proposed models (Model-0, Model-1, Model-2, and Model-3) validated by Kruskal–Wallis test, but also eight state-of-the-art approaches.

The shortcomings of our proposed Model-4 are threefold: (1) the model has not been verified clinically, but will certainly form the basis of future studies; (2) the model does not work with mammogram images, so we will aim to develop a hybrid model in the future which can help give predictive results regardless of whether the input is a thermogram image, a mammogram image or both.

The future direction will be following aspects: (1) try to expand the dataset and introduce more thermal images; (2) move our AI system online and allow radiologists worldwide to test our algorithm and (3) test other advanced AI algorithms.

## Appendix A

See Table 14.

**Table 14:** Abbreviation list

Abbreviation	Full meaning
DCIS	Ductal carcinoma in situ
BT	Breast thermography
BCS	Breast-conserving surgery
FE	Feature engineering
RL	Representation learning
IR	Infra-red
CCD	Charge coupled device
CMOS	Complementary metal-oxide–semiconductor
EC	Emitted component
VC	Vasoconstriction
VD	Vasodilation
PCM	Pseudo color map
CRLW	Compression ratio of learnable weights
NLDS	Nonlinear downsampling
RWP	Rank-based weighted pooling
LDA	L-way data augmentation
SSDP	Small-size dataset problem
CC	Color channel
GBL	Gradient-based learning
HS	Horizontal shear
VS	Vertical shear

## Appendix B

Suppose the input is *t*, traditional AF is in the form of sigmoid function $f_{{{\text{sig}}}}$, defined as

41 $$ f_{{{\text{sig}}}} \left( t \right) = \frac{1}{1 + \exp ( - t)}, $$

with its derivative as

42 $$ f^{\prime}_{{{\text{sig}}}} = f_{{{\text{sig}}}} \left( t \right) \times \left[ {1 - f_{{{\text{sig}}}} \left( t \right)} \right] $$

Sigmoid output is in the range of $\left[ {0,1} \right]$. In some situations, the range $\left[ { - 1,1} \right]$ is expected. $f_{{{\text{sig}}}} \left( t \right)$ could be shifted to become the hyperbolic tangent (HT) function

43 $$ f_{{{\text{HT}}}} \left( t \right) = \frac{{\exp \left( t \right) - \exp \left( { - t} \right)}}{\exp \left( t \right) + \exp ( - t)}, $$

with its derivative as

44 $$ f^{\prime}_{{{\text{HT}}}} \left( t \right) = 1 - f_{{{\text{HT}}}}^{2} \left( t \right). $$

Nonetheless, the widespread saturation of $f_{{{\text{sig}}}}$ and hyperbolic tangent function $f_{{{\text{HT}}}}$ make gradient-based learning (GBL) and its variants perform poorly in the neural network training phase. Hence, rectified linear unit (ReLU) $f_{{{\text{ReLU}}}}$ has grown in popularity, because it accelerates the convergence of GBL compared to $f_{{{\text{sig}}}}$ and $f_{{{\text{HT}}}}$.s

When $t < 0$, the activation of $f_{{{\text{ReLU}}}}$ values are zero, so ReLU cannot learn via GBLs, because the gradients are all zero. The leaky ReLU (LReLU) could ease this problem caused by changing hard-zero activation of ReLU. LReLU’s function $f_{{{\text{LReLU}}}} \left( t \right)$ is defined as

45 $$ f_{{{\text{LReLU}}}} \left( t \right) = \left\{ {\begin{array}{*{20}l}  {\beta \times t} \hfill &\quad {t \le 0} \hfill \\  t \hfill &\quad {t > 0} \hfill \\  \end{array} } \right., $$

where parameter $\beta = 0.01$ is the commonly pre-assigned value. Its derivative is defined as

46 $$ f_{{{\text{LReLU}}}}^{\prime } \left( t \right) = \left\{ {\begin{array}{*{20}l}  \beta \hfill &\quad {t \le 0} \hfill \\  t \hfill &\quad {t > 0} \hfill \\  \end{array} } \right.. $$

## Appendix C

Using Fig. 7 as an example, and assuming the region ${\Phi }\left( {1,1} \right)$ at 1st row 1st column of the input AM *I* is chosen as $\Phi \left( {1,1} \right) = I\left( {{\text{row}} = 1,{\text{col}} = 1} \right)$, the row vector of ${\Phi }\left( {1,1} \right)$ is $${\text{vec}}\left[ {{\Phi }\left( {1,1} \right)} \right] = \left( {\begin{array}{*{20}c} 8 & {0.2} & {2.2} & {1.1} \\ \end{array} } \right)$$. We can calculate the results of L2P is: $y_{{\Phi \left( {1,1} \right)}}^{{{\text{L}}2{\text{P}}}} = {\text{sqrt}}\left( {\left( {8^{2} + 0.2^{2} + 2.2^{2} + 1.1^{2} } \right)/4} \right) = {\text{sqrt}}\left( {\left( {64 + 0.04 + 4.84 + 1.21} \right)/4} \right) = 4.19$. The AP result is: $y_{{\Phi \left( {1,1} \right)}}^{{{\text{AP}}}} = {\text{average}}\left( {\Phi \left( {1,1} \right)} \right) = \left( {8 + 0.2 + 2.2 + 1.1} \right) \div 4 = 2.88$. MP result is: $y_{{\Phi \left( {1,1} \right)}}^{{{\text{MP}}}} = \max \left( {\Phi \left( {1,1} \right)} \right) = \max \left( {8,0.2,2.2,1.1} \right) = 8$. For the RWP, we first calculate the rank matrix is $${\text{vec}}\left( R \right) = \left( {\begin{array}{*{20}c} {r_{11} } & {r_{12} } & {r_{21} } & {r_{22} } \\ \end{array} } \right) = \left( {\begin{array}{*{20}c} 1 & 4 & 2 & 3 \\ \end{array} } \right)$$. Thus, $$\begin{array}{*{20}c} {{\text{vec}}\left( E \right) = \left( {\begin{array}{*{20}c} {e_{11} } & {e_{12} } & {e_{21} } & {e_{22} } \\ \end{array} } \right) = \left( {\begin{array}{*{20}c} \frac{1}{2} & {\frac{1}{{2^{4} }}} & {\frac{1}{{2^{2} }}} & {\frac{1}{{2^{3} }}} \\ \end{array} } \right)} \\ \end{array}$$. Finally, the RWP result is calculated as $y_{{\Phi \left( {1,1} \right)}}^{{{\text{RWP}}}} = \frac{8}{2} + \frac{0.2}{{2^{4} }} + \frac{2.2}{{2^{2} }} + \frac{1.1}{{2^{3} }} = 4.70$.
